# Micro and nanobubbles enhanced ozonation technology: A synergistic approach for pesticides removal

**DOI:** 10.1111/1541-4337.70133

**Published:** 2025-02-10

**Authors:** Preeti Pal, Arata Kioka

**Affiliations:** ^1^ Department of Systems Innovation, School of Engineering The University of Tokyo Tokyo Japan

**Keywords:** chemical free, nanobubbles, ozone treatment, residual pesticides

## Abstract

Pesticides production, consumption, and disposal around the world are raising concerns day by day for their human and environmental health impacts. Among developing treatment technologies, ozonation has attracted the attention of many researchers in recent years. It is an emerging and promising technology for removing pesticides in the aqueous environment and degrading the residual pesticides from the fruits and vegetables (F&V) surfaces. This systematic review presents an extensive study of the degradation of different types of residual pesticides from F&V using ozonation, micro‐ and nanobubble (MNB) ozonation, or other advanced techniques such as microwaves/ultrasonication and advanced oxidation process. This review compiles the studies that reported the effect of MNB size on the dissolution of ozone gas in the washing medium and its effect on the degradation of residual pesticides from F&V. The mechanism and routes of pesticide degradation and how integrating MNB technology (MNBT) can help overcome economic losses, reduce health issues for consumers, and save the environment from harmful chemicals used in the pesticides are also discussed. The article encourages the development and utilization of MNBT not only in agriculture, but aquaculture, fisheries, food industries, food storage, and packing, for reducing/degrading the residual pesticides from foods and support environmental sustainability as well as improve international trade.

## 1 INTRODUCTION

**FIGURE 1:**
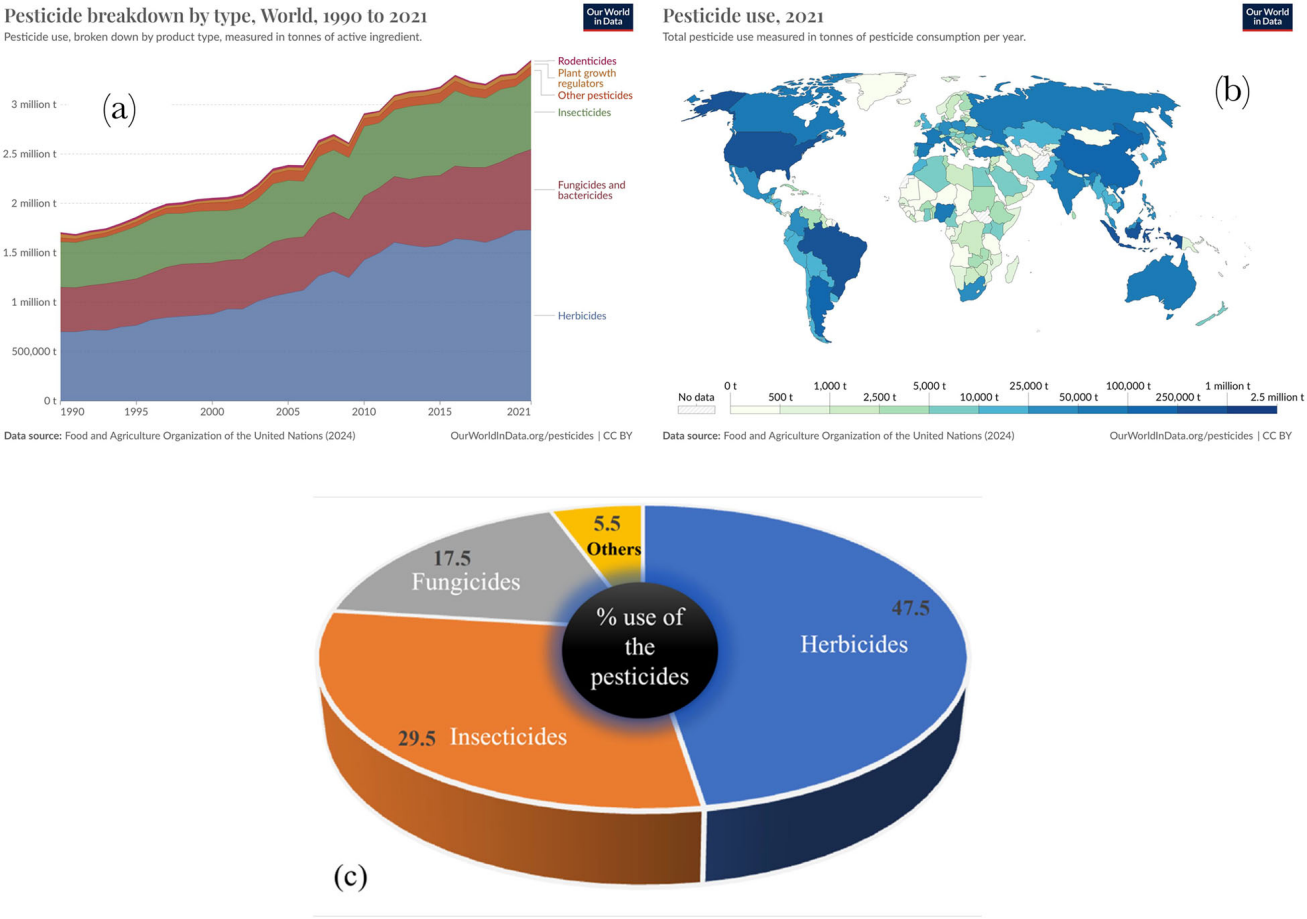
Gradual increase in the use of pesticides in past 31 years from 1990 to 2021 (in tonnes) as per their category (a) pesticides usage worldwide per year (b and c) usage of type of pesticides in percent (%).

Agricultural pesticides have been beneficial in increasing the yield and production of food. These pesticides can be used to overcome various kinds of threats to crops. Pesticides are commonly identified by the functional class of their active ingredient and the target organism they aim to control. Most of them can be categorized as insecticides, herbicides, fungicides, rodenticides, bactericides, and nematicides. Other chemicals primarily used in agriculture are growth regulators, plant defoliants, and surface disinfectants. As per the reports published by Statista Research Development (SRD) in 2024, global agricultural consumption of herbicides reached around 2.3 million metric tons; however, other pesticides all together were consumed less than one million. The use of herbicides alone will be expected to increase to around 2.4 million metric tons in 2027. It was reported that the overall use of pesticides in 2019 includes 49.5% herbicides, 29.5% insecticides, 17.5% fungicides, and 5.5% other pesticides (De et al., 2014) (Figure 1). SRD (2024) also reported that Brazil was the largest pesticide‐consuming country worldwide (800.65 thousand metric tons) followed by the United States (467.39 thousand tons). Between 1990 and 2021, global pesticide consumption increased by 96%, estimated at around 3.53 million metric tons (Hannah Ritchie et al., 2022).

Intensive use of pesticides is the very reason for residual pesticides on fruits and vegetables (F&V), and other food grains. Along with the direct exposure chemicals in the fields, chemical residues remain on the food material after harvesting can pose significant health risks to consumers (Alavanja Michael, 2009). However, they can also undergo volatilization, photolysis, or chemical and microbial degradation but the health risks associated with the pesticides have given rise to a sophisticated food safety industry that focuses on handling, preparing, and storing food in ways that prevent spoilage and food‐borne illness. In Japan, the Ministry of Health, Labor and Welfare (MHLW) has established a uniform limit of 0.01 mg L^−1^ for agricultural chemicals to ensure that the estimated intake does not exceed 1.5 µg day^−1^ based on the food consumption of the Japanese population. This decision aligns with the European Union's (EU) adoption of a similar uniform level in January 2005 as part of the positive list system. This not only applies to agricultural products but also to animal products or seafood (MHLW, 2006). With the United Nations Population Division anticipating a global population of 9.7 billion by 2050, the need for increased food production is pressing. Pesticides remain crucial in averting substantial crop losses, yet their use should be balanced with the need to protect human health and the environment by adhering to good agricultural practices. Farmers are encouraged to minimize pesticide usage to the essential amount and more rely on the produce without pesticides (WHO, 2023). Nearly, 600 agricultural pesticides are considered to be negatively affecting agricultural crops in terms of quality, and they cannot be marketed when they contain pesticides exceeding the residual limit (European Food Safety Authority (EFSA) et al., 2024; Rapid Alert System for Food and Feed [RASFF] Annual Report, 2021). Table 1 provides the brief of types of pesticides specifically used for F&V along with their residual concentration.

**TABLE 1:** Some examples of types of pesticides used for specific fruits or vegetables (F&V).

Type of crop	Pesticide type or broad name	Active compound	Functional route and effective organism	Effective against	MRL (EU) (mg kg^−1^)
Citrus fruit peels	Fungicide	Thiabendazole	Inhibition of Microtubule Formation: and disrupt cell division and intracellular transport in the pests. Inhibits the growth and reproduction in fungi (Naserzadeh et al., 2019; Zhou et al., 2011)	Fungal diseases	7.0
Nuts and nut products					0.02
Citrus fruit	Fungicides and disinfectants	Ortho‐phenyl‐ phenol (OPP)	Disruption of cellular membranes and protein denaturation (Hosoya et al., 2019)		10–15 (FAO, 1999)
Apples	Fungicide	Boscalid	Disrupt the energy metabolism of the fungus (Qian et al., 2018)		2.0
Potato	Organochlorine (I)	DDT	Disruption of nerve impulse transmission enzyme inhibition (Ecobichon, 1998)	Variety of insects	0.05
		Aldrin/Dieldrin			0.01
		Hexachlorobenzene, o.p‐DDD, p.p‐DDD			0.01
Tomato and egg plant	Organophosphorous (I)	Chlorpyrifos	Nerve and muscle disruption because of disruption in acetylcholine esterase which causes accumulation of acetylcholine and causes paralysis and death (Singh et al., 2023)	Aphids, thrips, and beetles, spider mites, various caterpillars, moths, leafhoppers	0.01
		Chlorpyrifos‐methyl			0.01
		Malathion			0.02
Egg plant		Profenofos			0.01
Seeds of tomato	Organophosphate (I)	Dimethoate			0.01
		Profenofos			10
Apples		Malathion			0.02
Apples		Chlorpyriphos and Chlorpyriphos‐methyl			0.01
Peppercorn (black, green and white)		Chlorpyriphos and Chlorpyriphos‐methyl			0.01
Peaches		Acephate, Chlorpyriphos and Chlorpyriphos‐methyl			0.01
Rapeseed/canola seeds		Chlorpyriphos, Chlorpyriphos‐methyl			0.01
Oilseeds		Malathion			0.02
Rapeseed		Pirimiphos‐methyl			0.5
Peaches	Phosphorothioate (I)	Fenitrothion			0.01
Raspberry	*Saccharopolyspora spinosa*	Spinosad	Effect the nervous systems and cause the insect's muscles to flex uncontrollably, which leads to paralysis and death (Santos & Pereira, 2020)	Fire ants, fruit flies, leafminers, mites, mosquitoes, spider mites, and thrips	1.5
Sunflower	Organochlorine (I)	Endosulfan	Disruption of nervous system function in insects which cause overstimulation and paralysis (Santos & Pereira, 2020)	Aphids, fruit worms, beetles, leafhoppers, moth larvae, and white flies	0.1 (Bellisai et al., 2023)
Tomato	Fungicide	Difenoconazole	Inhibition of demethylation during ergosterol synthesis	Fungal disease control	0.05 (Denis J. Hamilton, 2007)
Strawberry	Acaricide (A)	Hexythiazox	Control the growth of mites by acting on their eggs	Mites	0.05
	Herbicide (H)	Alachlor	By disrupting their growth cycle	Grasses and broadleaf weeds	0.05
	Organophosphate (I)	Acephate	Disruption of Nervous System Function in insects	Aphids, fruit worms, beetles, leafhoppers, moth larvae, and white flies	0.05
	Insecticide	Aldrin and Dieldrin			0.02
Chinese Cabbages	Anthranilic diamides (I)	Chlorantraniliprole	Causes ryanodine receptor Activation which disrupt the calcium channels and causes muscle contraction disruption due to excessive release of calcium ions in the muscles (Cordova et al., 2006)	Caterpillars, moth, butterflies	20
Lettuces and salad plants	Fungicide	Azoxystrobin	Inhibits spore germination, mycelial growth, and the spore production in fungi (http://npic.orst.edu/)	Fungal disease control	10.0
	Insecticide	Deltamethrin, Imidacloprid	Disrupt the normal nervous system function	Caterpillars, moth, butterflies	0.01
	Herbicide	Glyphosate	Acts by inhibiting the plant enzyme 5‐enolpyruvylshikimate‐3‐phosphate synthase (http://npic.orst.edu/)	Grasses and broadleaf weeds	0.1
Peaches	Fungicide	Fludioxonil	Inhibits fungal signal transduction pathways, disrupting cell division and growth (Zhou et al., 2011)	Controls fungal diseases such as botrytis and mildew	10.0
Orange		Imazalil			4.0
Pears		Pyrimethanil	Fungicide that inhibits fungal cell wall synthesis	fungal pathogens causing brown rot or other diseases	15.0
Vegetable, legume, edible‐podded groups		Boscalid	It inhibits fungal succinate dehydrogenase, which impairs energy production in fungal cells (Masiello et al., 2019)	Various fungal cells	3.0
Spinach	Pyrethroid insecticide	Permethrin	Disrupts the nervous system of insects by binding to sodium channel	Aphids, flea beetles, and caterpillars, leafminers, and spider mites	0.05
		Cypermethrin			0.7
Rapeseed		Deltamethrin			0.02
Grapes	Neonicotinoid insecticide	Imidacloprid	binding to nicotine acetylcholine receptors in insects, leading to overstimulation, paralysis, and death due to disrupted nerve signal transmission. (Ihara & Matsuda, 2018)	Aphids, whiteflies, and mealybugs	0.7

Abbreviation: MRL, maximum residue limit.

*Source*: EU: https://ec.europa.eu/food/plant/pesticides/eu‐pesticides‐database/start/screen/mrls data retrieved from EU Pesticides Database, 2022 update.

**FIGURE 2:**
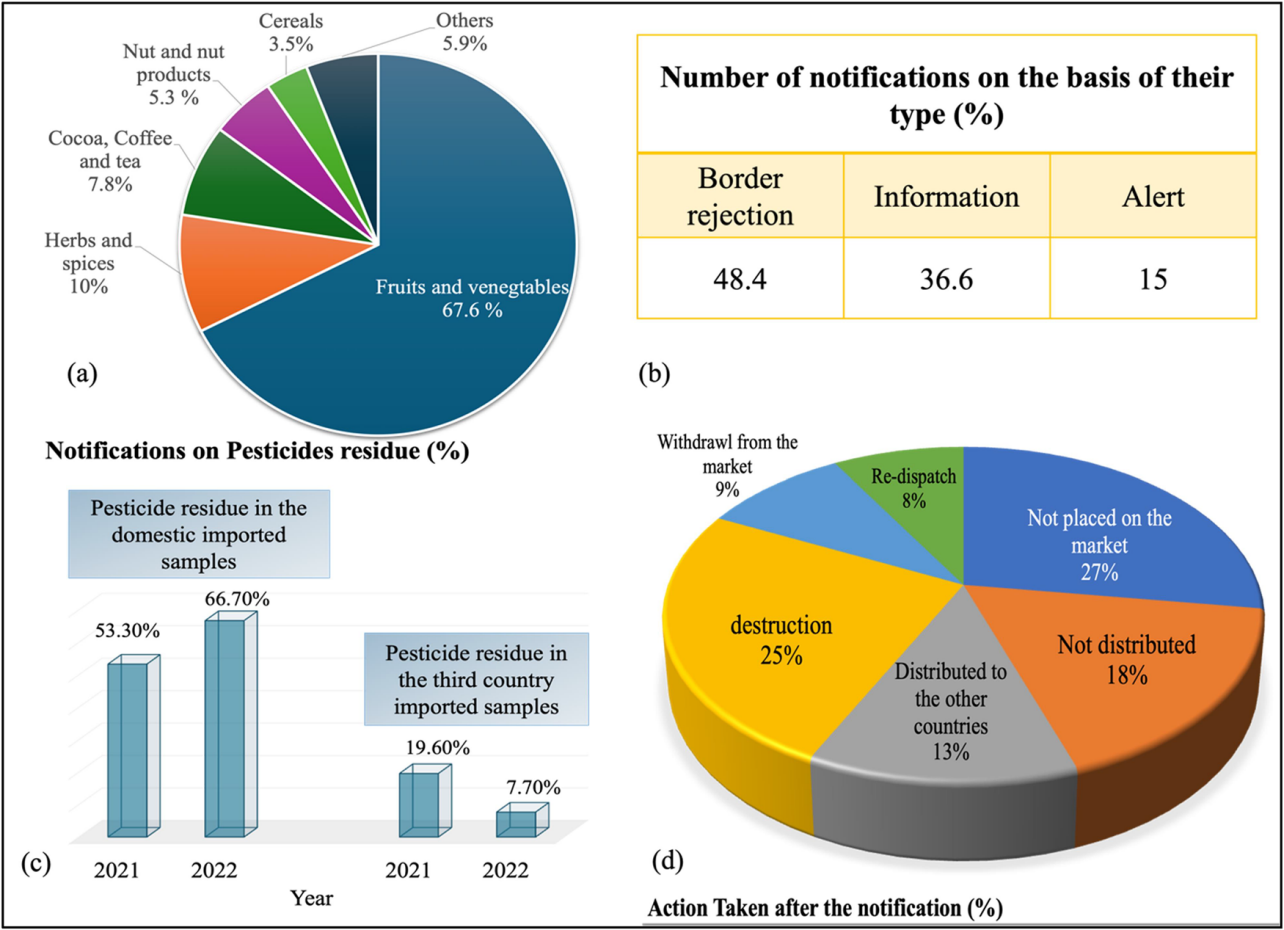
Summary of some selected data on the basis of variable such as category of food (a), type of notification (b), pesticides residues found in domestic and internation samples comparison of 2021 and 2022 (c), actions taken by the respective authority after generation of notification (d).

**FIGURE 3:**
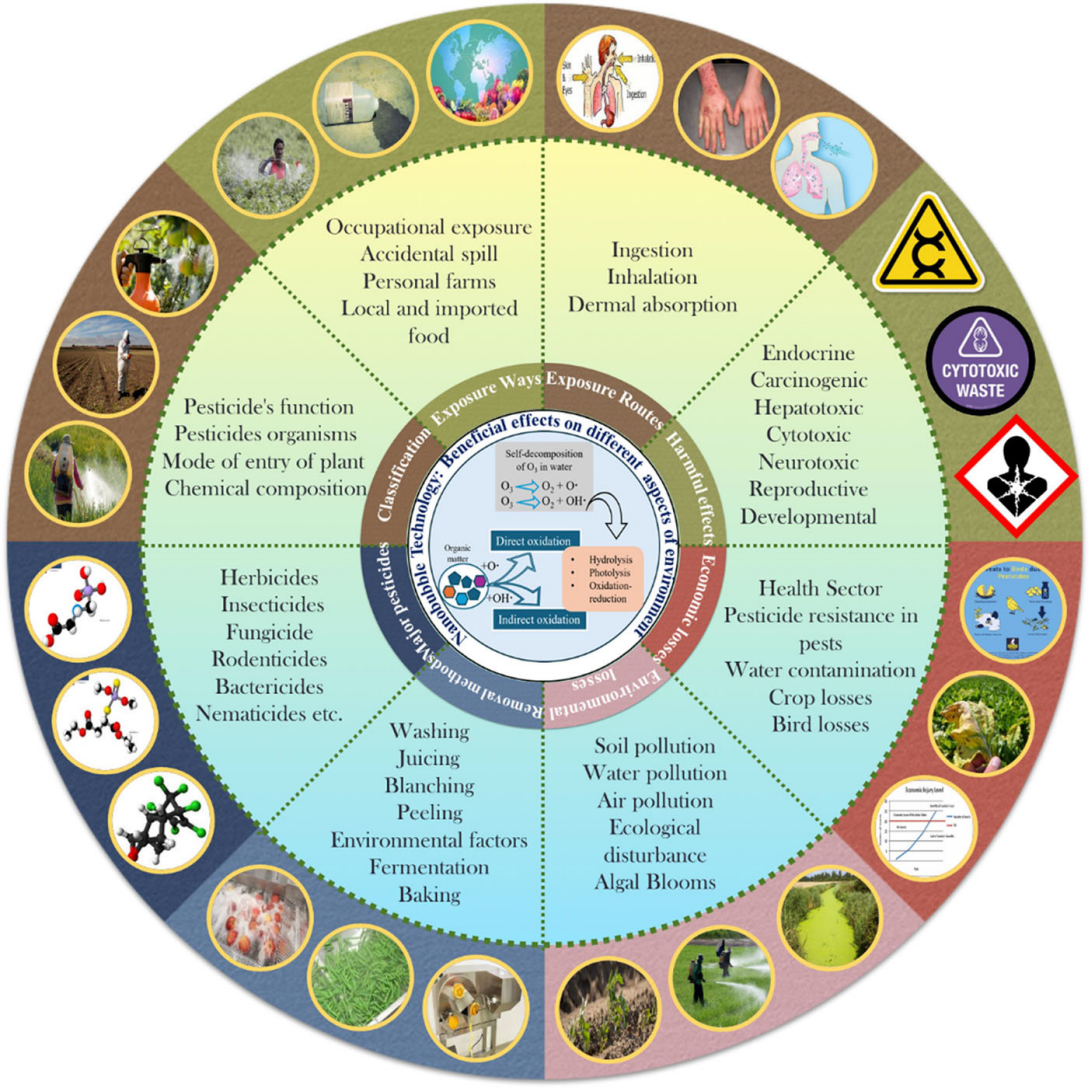
Different aspects to study pesticides and their effect on environment and human health.

According to EU's RASFF, which is involved in finding and tracing down the contamination in food and their distribution, the most notified category was F&V (RASFF Annual Report, 2021). Pigłowski (2022) reported that pesticide residues suddenly rank second in the Top 10 hazards for food products. In 2020, ethylene oxide (347 notifications), followed by chlorpyrifos (48 notifications), pyridaben (43 notifications), and chlorpyrifos‐methyl (41 notifications) were top in the list of food contaminants (Pigłowski, 2022; RASFF Annual Report, 2021). The data of RASFF from 1981 to 2020 identify the most frequently notified pesticides considering different factors such as the notifying year, food category, origin, and notifying counties and actions taken (Figure 2). F&V, herbs, and spices were the most contaminated by pesticide residues and resulted in rejections. These rejections result in huge food loss; for example, the products that usually came from India or Turkey were not placed on the market or were not distributed and then destroyed. The data from 4061 notifications in the period 1994–2020 suggested that nearly half of all notifications (48.4%) were border rejections, 36.6% were information notifications and alerts only 15.0% (Figure 2b). Major notifications were related to F&V, which were 67.6%; herbs and spices 10.0%; cocoa, coffee, and tea 7.8%; nuts, nut products, and seeds 5.3%; cereals 3.5%; and others 5.9%. Overall, 25% and 27% of these food products were destructed and not placed in the market, respectively (Figure 2d). The originated country for these products were mainly India (18.1%), Turkey (17.6%), China (7.8%), Thailand (6.2%), Egypt (5.8%), and Italy (3.7%) (Pigłowski, 2022). EFSA et al. (2024) reported in that residual pesticides concentration in domestic imports increased from 53.3% to 66.7% from 2021 to 2022, whereas that in imports from third country reduced from 19.6% to 7.7% (Figure 2c). There is a need to develop effective methods for pesticide degradation, which causes health issues, environmental contamination, and economic losses during exports. Figure 3 gives a summary of effect of pesticides on environment and human health which suggests the importance of degrading the pesticides to prevent it from human consumption.

There are various processes involved after harvesting to process the food before consumption to make it free from chemicals and other contaminants. Washing, peeling, juicing, blanching, fermentation, baking, ozonation, ultrasonication (US), and advanced oxidation are some of the main food processing techniques applied to F&V products before consumption. Here, we have tried to emphasize one of the effective treatment. Many studies reported the use of ozone (O_3_) as a potent candidate for removing various contaminants, including pesticides and insecticides either in combination with other techniques or independently. O_3_, a natural substance in the atmosphere, is one of the powerful oxidizing agents working as a sanitizer against a wide variety of microorganisms (Vuthijumnonk & Shimbhanao, 2019) for the prevention of waterborne and airborne diseases. Furthermore, O_3_ is considered nonhazardous, because, in aqueous solution, it auto‐decomposes quickly to convert back to oxygen. However, the instability of O_3_ in aqueous media is one of the main limitations of O_3_ treatment which can be improved by reducing the size of bubbles before mixing it into water. Air micro‐ and nanobubbles (MNBs) can improve gas–liquid contact and help to achieve increased effectiveness by enhanced mass transfer (Seridou & Kalogerakis, 2021).

O_3_ is also known as activated oxygen due to its extra oxygen atom, which preserves the flavor of food by its autolysis to oxygen and extending shelf life of the fruits (Ikeura et al., 2011a, 2011b; Lozowicka & Jankowska, 2016). Hence, MNB aqueous O_3_ can offer a viable solution to all the problems associated with pesticides in the agricultural system. MNB technology (MNBT) emerges as a promising solution for addressing climate change and environmental issues, reducing costs and energy consumption in industries, optimizing therapeutic and diagnostic techniques, and various other applications. The present work focuses on the removal of residual pesticides by various advanced methods, with a specific emphasis on the O_3_ MNB treatment method. We have reviewed recent papers utilizing this novel MNB treatment method to deal with the residual pesticide problem. Despite being a relatively recent development, there is a wealth of reports and studies highlighting the properties of nanobubbles (NBs) and their potential in diverse sectors. This review aims to provide concise information on recent scientific findings regarding the versatility and sustainability of NBs for pesticides and residual pesticide removal and how this affects the economy of producers and consumers. The paper also discusses the limitations of implementing the newly developed methods in in vivo conditions. To overcome the economic issues associated with pesticide use, we need to invent and adopt new chemically‐benign, cost‐effective, and environmentally friendly technologies that can efficiently reduce or eliminate residual pesticides from food products before supplying them to the market. The long‐term sustainability of agriculture is always a concern when hazardous chemicals are used. By such technologies not only agriculture but food processing and packing industries, dairy industries, meat processing, and storage, cleaning, sanitation will also be benefitted.

## 2 ADVANCED METHODS FOR PESTICIDES REMOVAL

F&V undergo culinary and food processing procedures prior to consumption. The impact of these handling methodologies on pesticide residue levels in F&V can be influenced by the physical distribution of the residues and the physico‐chemical attributes. Encompassing solubility, volatility, hydrolytic rate constants, water‐octanol partition coefficient, and thermal degradation are some of the factors that affect the removal/degradation efficiency of the processing methods. Primary food processing techniques for F&V products before consumption include washing, peeling, juicing, blanching, fermentation, and baking. Washing and peeling emerge as the most efficient method in combination with the novel technologies for diminishing residual pesticide levels before consumption. Heshmati and Nazemi (2018) investigated new methods to reduce dichlorvos (DDVP) residues on tomatoes postharvest along with the washing. They reported that washing with tap water, ozonated water (2–6 mg O_3_ L^−1^, in total 2 L solution), detergent solution (1%–3% concentration), and ultrasonic cleaning (100–300 W) significantly reduced DDVP levels. Maximum removal percentages after 15 min were as follows: tap water (30.7%), ozonated water (91.9%), detergent solution (70.7%), and ultrasonic cleaner (88.9%). For generating O_3_ fine bubbles, O_3_ was diffused through a stainless‐steel filter. These methods effectively lowered DDVP residues without compromising tomato quality, offering strategies to mitigate dietary exposure risks. Immersing tomato samples for 30 min in tap water and detergent water (conc.: 0.25% and 1%) resulted in 16%–44%, 27%–52%, and 38%–61% removal, respectively, whereas the application of gaseous O_3_ led to a 2.8‐fold greater reduction in azoxystrobin fungicide (Rodrigues et al., 2019).

Although current methods are effective, there is still much to improve. Juicing, for example, involves the extraction of liquids from F&V, often assisted by enzymatic treatment to enhance juice yield. Throughout the juicing process, a combination of additional steps such as washing, pressing, sterilization, and enzymatic treatment significantly contributes to reducing pesticide residues in the matrix. Li et al. (2015) studied the effect of food processing steps on pesticide residues in apples and investigated the residue levels of beta‐cypermethrin, chlorpyrifos, tebuconazole, carbendazim, and acetamiprid. Their results elucidated that the juice processing led to an 85%–95% decrease in pesticides residues in apple matrices (Li et al., 2015). Baking can also be helpful in reducing or diminishing pesticide residues through evaporation or degradation by heat. The storage of F&V before processing can also play a significant role in the degradation of pesticide residues over time, although the extent of degradation varies depending on the active ingredient. It is reported that storage conditions will impact the fate of residues and the degradation of pesticides through storage, depending on the type of condition in which they are stored. For instance, the presence of the fungicide dodin and insecticide phosalone in apples was detected after 5 months of cold storage at 1–3°C, whereas the other pesticide azinphos‐ethyl has 10 days half‐life on trees and 83 days for apples stored at ambient conditions (18 ± 5°C, RH ∼60%) (Nguyen et al., 2020).

**FIGURE 4:**
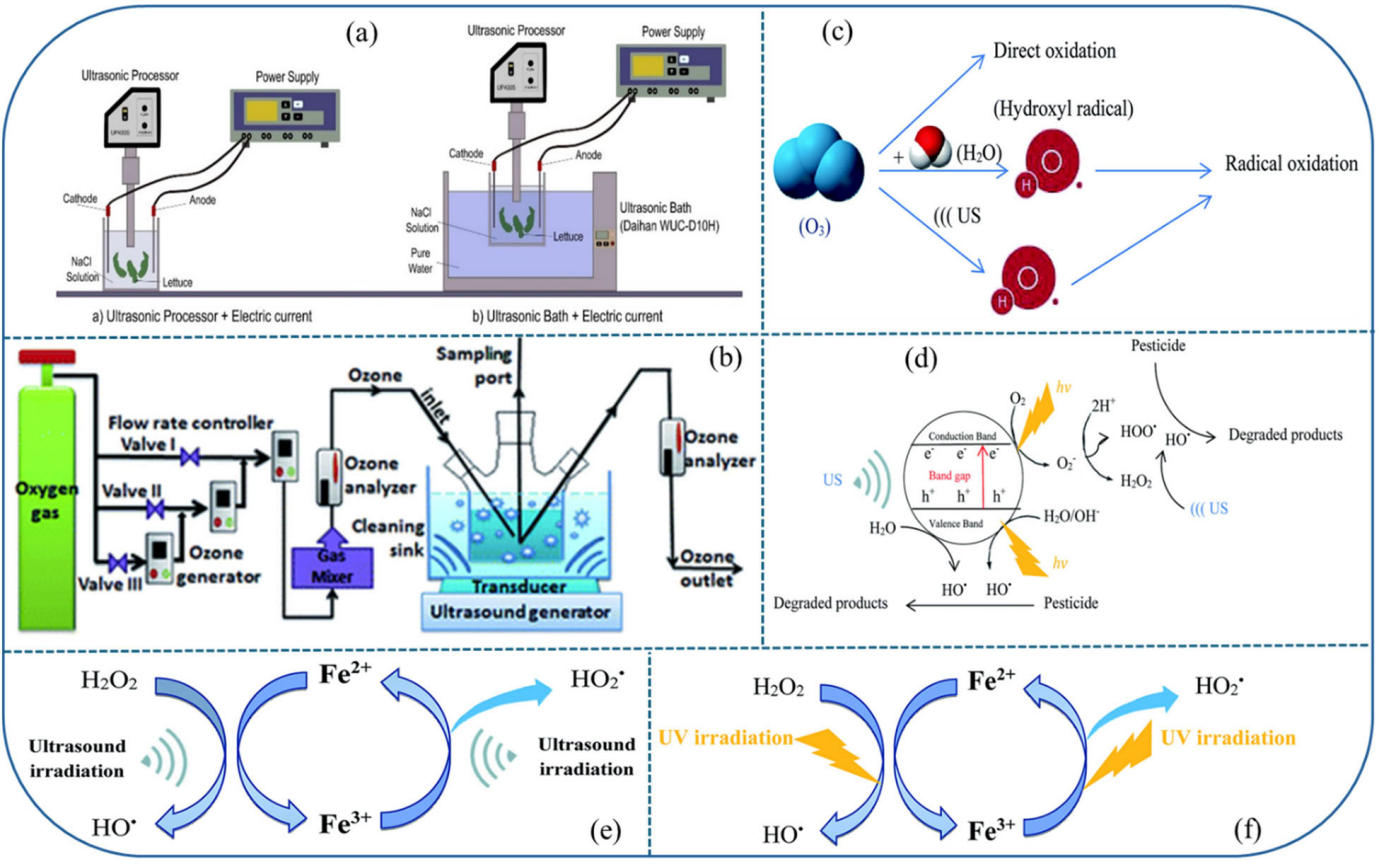
Advanced methods available for the removal of residual pesticides: (a) Ultrasonication (US) combined with electric current (Cengiz et al., 2021); (b) schematic of experimental US/O_3_ combination cleaning apparatus (Fan et al., 2015); (c) Proposed schematic ultrasound/ozonation mechanism; (d) schematic sono‐photocatalytic degradation mechanism of pesticides; (e) sono‐Fenton process; (f) sono‐photo‐Fenton process (Pirsaheb & Moradi, 2020).

The future of food safety looks promising with the emergence of advanced techniques such as sonozonation (Taiye Mustapha et al., 2020), chlorination, US (Yang et al., 2022), sonochemical degradation (Pirsaheb & Moradi, 2020), ozonation (AlAntary et al., 2018; Díaz‐López et al., 2021; Martínez‐Escudero et al., 2022), and solarization (Garrido et al., 2023; Martínez‐Escudero et al., 2022), which are gaining popularity in the research field. Ultrasound effectively degrades pesticides through pyrolysis and free radical reactions, influenced by frequency, power, initial pesticide concentration, temperature, pH, and dissolved gas. Yang et al. (2022) explored a novel advanced oxidative process (AOP), developing the combined free chlorine/ultrasound method to remove three common pesticides from lettuce. The free chlorine/ultrasound process achieved removal efficiencies of 86.7% for dimethoate (DMT), 79.8% for trichlorfon (TCF), and 71.3% for carbofuran (CBF). The study found a synergistic effect in the coupled free chlorine/ultrasound process, with synergistic factors of 22.3% for DMT, 19.0% for TCF, and 36.4% for CBF, indicating enhanced pesticide removal efficiency (Yang et al., 2022). It is evident that chlorine helps breaking down complex molecules through oxidation and also forms hypochlorous acid (HOCl), which is an even stronger oxidant and effective in degrading a wide range of organic pollutants (Cengiz & Certel, 2014). Despite promising lab results, scaling up for industrial use requires further research on kinetics, reactor design, cost‐effectiveness criteria, and feasibility in large‐scale applications. There have been limited studies exploring the integration of electrocoagulation with AOP for pesticide removal, specially in effluent water, particularly concerning O_3_ and electro‐Fenton methods. Hence, future research should prioritize evaluating the efficacy of these combined approaches in treating pesticide‐containing effluents (Pirsaheb & Moradi, 2020). Some of the selected methods are depicted in Figure 4, which can be applied to enhance the degradation of residual pesticides in F&V. Such methods involve US combined with electric current (Cengiz et al., 2021), US/O_3_, (Fan et al., 2015), sono‐photocatalytic degradation, sono‐Fenton process, and sono‐photo‐Fenton process (Pirsaheb & Moradi, 2020).

Advanced methods combine US, light, and chemical reaction to degrade the organic pollutants. For instance, sono‐photocatalytic degradation involves the combination of sonochemistry and photocatalysis (to activate a catalyst) to degrade organic pollutants (Figure 4d). In principle, sonication refers to the creation of high‐frequency sound waves to change the pressure in the liquid which collapses the tiny bubbles and causes cavitation. This produces highly reactive hydroxyl radicals (•OH) which helps in the degradation of pollutants. Photocatalysts (such as TiO_2_/ZnO/CuO/FeO) when activated by ultraviolet (UV) or visible light, the excited electrons generate electron‐hole (e^−^‐h^+^) pairs that can produce highly reactive species (•OH) to degrade organic matter. The combination of US and light synergistically enhances the generation of radicals and increases the degradation efficiency. On the other hand, the process which completely relies on natural solar light for the activation of photocatalysts is referred as solarization often used in conjunction with photocatalysis or other AOPs to degrade organic contaminants (Garrido et al., 2023; Martínez‐Escudero et al., 2022).

The sono‐Fenton process combines US and the traditional Fenton reaction. In combination, US facilitates the decomposition of hydrogen peroxide (H_2_O_2_) into hydroxyl radicals (•OH) in presence of catalyst (Fe^2+^ + H_2_O_2_ → Fe^3+^ + •OH) and enhances the degradation by the sono‐Fenton reaction (Figure 4e). Sono‐photo‐Fenton is a hybrid of the sono‐Fenton and photocatalysis processes, combining ultrasound, visible or UV light, and the Fenton reaction to achieve more efficient, rapid, and cost‐effective pollutant degradation (Figure 4f). The combination creates a powerful degradation system that leads to a much higher efficiency than any of the individual methods alone (Pirsaheb & Moradi, 2020).

Sonozonation is the combination of US and ozonation (O_3_) for the degradation of pollutants in water and wastewater. The combination of both the techniques enhances the production of reactive oxygen species (ROS), improving the overall efficiency of degradation. O_3_, when introduced into the water, decomposes into •OH and other ROS under certain conditions (Xiong et al., 2019) (Figure 4b). The synergy between O_3_ and US leads to faster decomposition of pollutants (Taiye Mustapha et al., 2020). During US, the physical effects of sonication can also improve the diffusion of pollutants into the reaction zones, increasing the degradation rate by enhanced mass transfer (Yang et al., 2022). Similarly, US combined with the electric current induces cavitation to generate high‐energy hotspots, whereas the electric current enhances electrochemical reactions, producing additional ROS (Cengiz et al., 2021). Some of the main advantages of the advanced processes are their increased efficiency because degradation of pollutants compared to conventional methods is faster. They are nontoxic and flexible to work on a wide range of organic pollutants. The following section compiles the studies that specifically focused on the use of O_3_ for pesticides removal.

### 2.1 Gaseous or aqueous ozone treatment for pesticide residue removal

**FIGURE 5:**
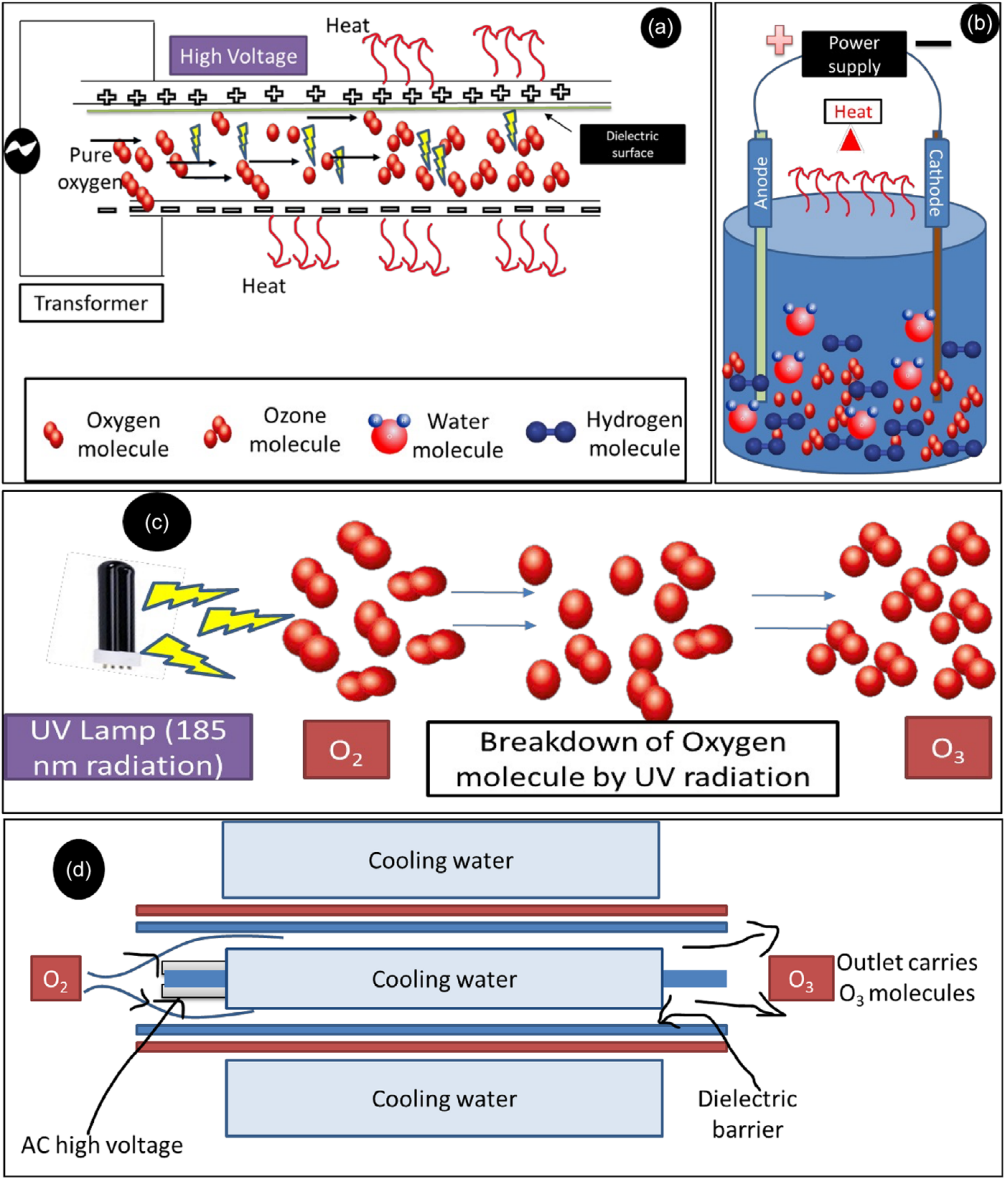
Pictorial presentation of common methods of O_3_ generation (a) corona discharge method, (b) electrolysis, (c) ultraviolet (UV) radiation, and (d) cold plasma technique.

O_3_ is a natural disinfectant used in hospitals, agriculture, sanitation, wastewater treatment, and potable water treatment. O_3_, a triatomic form of oxygen, has proven to be a versatile tool with applications in agriculture and sanitation (Prabha et al., 2015). Its significant efficacy as a versatile disinfectant and its ability to eliminate pesticide residues have led to its widespread use in enhancing soil quality, disinfecting food, and treating water. Its capability to break down pesticides without adverse environmental effects has further increased its acceptance as a method for post‐pesticide cleanup. When pesticides come into contact with O_3_, it decomposes and transforms them into the harmless end products, such as carbon dioxide, water, and other inert substances (Aidoo et al., 2023). The use of O_3_ in treating pesticide residues in soil, water, and food has proven to be highly successful. Recent advancements in pesticide residue removal methods are highlighted, along with a discussion on challenges associated with O_3_ treatment. Whether applied independently or combined with other techniques, O_3_ is a highly efficient means of eradicating pesticide residues from the environment (Pirsaheb & Moradi, 2020). O_3_ is recently reported as an effective disinfectant against SARS‐CoV as well during COVID 2019 pandemic (Cristiano, 2020; Franke et al., 2021; Grignani et al., 2020; X. Hu et al., 2021). O_3_’s stability in water is noteworthy; it exhibits a relatively short half‐life, ensuring its potent disinfection effects while minimizing environmental impact. However, the bottleneck includes that countries where the temperature goes as high as 43–45°C, O_3_ stability is lower, and the reaction speed increases with a factor of 2 or 3 per 10°C. Half‐life of O_3_ is less than 8 min at the temperature above 30°C; hence, it is necessary to increase the stability of O_3_ in aqueous medium for field applications (Pal & Anantharaman, 2022). This highlights a current challenge and potential topic for future research. Figure 5 schematically represents the most widely used methods for O_3_ generation for different purposes, and a summary of the methods is given in Table 2.

**TABLE 2:** Few popular methods of O_3_ generation and their brief summary.

Methods of O_3_ generation	Brief overview	O_3_ generation
**Corona discharge**	One of the most common methods Utilizes a high electrical charge diffused across a dielectric surface When oxygen passes through the resulting electric field, oxygen molecules are split into individual oxygen atoms which then bond with other oxygen molecules, forming O_3_ (Ozone Production Methods, n.d.; Rodríguez‐Peña et al., 2021)	7.53 mg L^−1^ with the voltage of 20 kV at 3 kHz frequency (Singhapuntu & Thungsuk, 2016) Upto 15% with the concentrated O_2_
**Electrolysis**	Electrolysis passes a current through water and splits the oxygen atom in water molecule which creates O_3_ gas, oxygen gas, and hydrogen gas requires pure water to be used Generates a lot of heat (Okada et al., 2019; Rodríguez‐Peña et al., 2021)	160 mg L^−1^ (Okada et al., 2019)
**UV irradiation**	Ultraviolet lamps emitting light at 185 nm is used Oxygen passes over the UV lamp and O_2_ molecules split into single oxygen atoms which bond with other O_2_ molecules to form O_3_ Non‐ efficient method Generates small amounts of O_3_ (Zoschke et al., 2014)	0.5% using Air and 1% using oxygen (Ozone Production Methods, n.d.)
**Cold plasma**	Expensive process Generates O_3_ by passing pure oxygen through cold plasma (Antipov et al., 2024)	Air flow of 0.4 Lmin^−1^, >1000 ppm O_3_ (Mohamed et al., 2004)

One of the methods of O_3_ generation is where air or oxygen gas is passed through a high voltage electrical discharge (corona discharge technology) or by UV light irradiation (Figure 5) (Favvas et al., 2021; Seridou & Kalogerakis, 2021) and then it is passed through the water for converting gaseous O_3_ into aqueous O_3_ in the form of microbubbles (MBs) or NBs. The MBs (10–100 µm diameter) and NBs (<1 µm) are very different in sizes which makes them completely different in terms of their properties (Aikawa et al., 2021; Kioka & Nakagawa, 2021; Nakagawa et al., 2022; Pal & Anantharaman, 2022; Katagiri et al., 2023). These bubbles of aqueous O_3_ create strong ·OH which acts as strong oxidative and can be used for sterilization, bleaching, virus inactivation, degradation of residual pesticides, organic matter, deodorization, and mycotoxin degradation (Megahed et al., 2019; Tekile et al., 2017). Various studies reported the use of O_3_ for residual pesticides removal from the F&V as well as a helpful tool from storage to consumption before and after harvest. For instance, Özen et al. (2021) studied the effects of O_3_ treatment for residual pesticides removal, increased storage life, and quality of green peppers. Pesticides, including malathion, emamectin benzoate, and acetamiprid, were sprayed on pepper plants before harvest and measured the residue substances of peppers before and after harvest time. O_3_ treatment was given with water and air as 2.0 mg L^−1^, and tap water and air were used as control. Treatment time for aqueous O_3_ was 10 min, whereas the air treatment was given for 45 min followed by storage of treated peppers at 20°C and 60% ± 5% relative humidity for 8 days. The ozonated water remarkably decreased the pesticide residues when compared to harvest time, but no significant changes were observed in the O_3_ air treated samples. Ozonated water can be an alternative treatment to extend storage life of green peppers and remove pesticide residues. It also maintained the green color of peppers with minimum change in h values (or spiciness/heat) (Özen et al., 2021).

Bae et al. (2023) tried three treatment methods for pepper which are UV, O_3_, and photochemical AOP (pAOP). The study reported that UV‐C (254 nm) and UV‐A (360 nm) treatment under 9.6 W m^−2^ of UV exposure for 24 h was able to reduce 59.7% and 13.3% residue concentrations, respectively. Residue concentrations up to 57.9% on average were reduced by gaseous O_3_ treatments, whereas pAOP treatment proved to be the most effective method by reducing the concentration up to 97%. A volume of 12 µmol mol^−1^ O_3_ was able to reduce over 50% of the pesticide residues from difenoconazole, fludioxonil, imidacloprid, and thiamethoxam. On the other hand, pesticides such as carbendazim, fluquinconazole, and pyrimethanil were relatively stable, and even after increasing the O_3_ concentration up to 24 µmol mol^−1^, they were only able to be reduced below 50% in pAOP treatment. Therefore, the authors suggested that pAOP treatment, when combined with the gaseous O_3_, can be helpful for significant reduction of the pesticide concentration during storage and can also increasing the shelf life without greatly reducing the quality of pepper (Bae et al., 2023).

Lemic et al. (2024) reported that O_3_ treatments have demonstrated remarkable effectiveness in reducing mold growth in citrus fruits. The gaseous O_3_ can achieve efficacy rates as high as 97.5%, whereas ozonated water treatments maintain preservation rates between 95% and 97% (Lemic et al., 2024). O_3_ treatments have also been shown to preserve the quality of fresh produce, including texture, visual appearance, taste, aroma, and nutritional content, without causing harmful effects. Treatments that can reduce microbial contamination without compromising the visual, textural, and nutritional attributes of the product are suitable for recommendation and integration into the supply chain (Glowacz et al., 2015). Fan et al. (2015) have shown the combined effect of US/O_3_ to degrade methamidophos and DDVP pesticides on lettuce. Their study achieved a degradation rate of up to 82.16%. Optimal conditions included an O_3_ flow rate of 75 mg min^−1^, a treatment time of 60 min, initial pesticide concentrations ranging from 0.1 to 0.2 mg kg^−1^, and a water temperature of 8°C. Importantly, the treatments did not negatively affect the quality of the lettuce, suggesting a promising method for pesticide residue reduction in agriculture (Fan et al., 2015). Many studies have come up with combining two or more technologies together to give better efficiency for removing pesticides. Such as combining O_3_/US, H_2_O_2_/O_3_, UV/H_2_O_2_/O_3_, or US/O_3_, among all US/O_3_ has proven highly effective in enhancing AOP for pesticide residue degradation (Cengiz et al., 2018; Fan et al., 2015; Taiye Mustapha et al., 2020). This approach minimizes energy consumption, avoids selective degradation, and prevents secondary pollution, making it superior to standalone methods like US or O_3_ alone. The synergy improves oxidant utilization by enhancing O_3_ dissociation and overcoming mass transfer limitations through increased turbulence. Moreover, it offers significant environmental benefits, aligning with the growing need for sustainable agricultural practices (Jia et al., 2023; Pirsaheb & Moradi, 2020).

Heleno et al. (2015) reported a comparison study between gaseous O_3_ and aqueous O_3_. Results depicted that gaseous O_3_ can result in 67.4% reduction, whereas aqueous O_3_ treatment achieved a 78.9% reduction in chlorothalonil residues in table grapes (Heleno et al., 2015). In a study involving lemon, orange, and grapefruit matrices, Kusvuran et al. (2012) observed varying adsorption levels of chlorpyrifos ethyl, tetradifon, and chlorothalonil depending on the type of fruit. They found that a 5‐min ozonation treatment completely eliminated chlorothalonil residues from the orange matrix. The lemon matrix exhibited the highest adsorption and diffusion rates for tetradifon. However, the study did not establish a clear relationship between the efficacy of pesticide removal and the structural properties of the fruit matrices despite O_3_ proving effective in removing all three pesticide residues from fruits, even from peeled surfaces (Kusvuran et al., 2012). However, wetting and spreading are of key importance for application of herbicides and pesticides on the plants (Bergeron et al., 2000). At large spatial scales, wetting or non‐wetting plays an important role in the efficient deposition of pesticides on plant leaves, F&V and influences the interaction of MNBs with the surfaces (Jia et al., 2023) and thereby the removal process of the residual pesticides. It is reported that highly diluted washing solutions may lead to rebounding from the surface of F&V due to the outer wax‐like layer which is said to bound with the pesticides and causes it to deposit on the surface and make it difficult to remove in the washing process (Guo et al., 2023; Song et al., 2023). Wu et al. (2019) compared various washing methods for removing 10 pesticide residues from spinach, kumquat, and cucumber. They tested tap water, O_3_ water, alkaline electrolyzed water, micron calcium solution, sodium bicarbonate, and active oxygen. Results showed that active oxygen was the most effective, completely removing all 10 pesticide residues due to its strong oxidizing and alkaline properties. O_3_ water also proved effective, removing 20%–40% more residues than tap water. The study emphasized that the efficacy of pesticide removal depends on treatment duration, pH of the washing solutions, and specific chemical properties of the pesticides (Y. Wu et al., 2019).

### 2.2 Micro‐ and nanobubbles (MNBs) ozone treatment

MBs are bubbles with typical diameters of 10–100 µm, whereas the size of NBs ranges in nanometers (Alheshibri et al., 2016; Pal & Anantharaman, 2022; Temesgen et al., 2017). Various types of generators can produce MNBs in water. Although the characteristics of MNBs can differ depending on the type of gas used, they share fundamental properties such as the generation of free radicals, which improves the oxidation capacity, self‐pressurization, and a negative surface charge/zeta potential, making them suitable to be used in various applications (Jia et al., 2023). The MNB technique has been documented for its effectiveness in removing insecticides (Baram et al., 2022; Takahashi et al., 2007; Vuthijumnonk & Shimbhanao, 2019). As the typical ozonation system does not produce adequate bubble size and is not stable in a water medium, as mentioned earlier, MNB aqueous ozone (MNBO) systems based on MNBT provide a natural alternative solution to chemicals and pesticides. NB system generates gas bubbles in the aqueous medium which is a mixture of millions of MNBs, filled with gas, and have longevity (Batagoda et al., 2018). Nirmalkar et al. (2018) stated that these nano‐entities can remain stable for months without altering the diameter. There is no bubble amalgamation, breaking, or Ostwald ripening (Nirmalkar et al., 2018). The zeta potential of the NB in the neutral‐to‐alkaline aqueous solution is generally highly negative, preventing them from coalescing (L. Hu & Xia, 2018; Kioka & Nakagawa, 2021; Nirmalkar et al., 2018). The important property of NBs is that they stay in water for longer periods and overcome buoyancy, whereas millibubbles or MBs rise. Properties, such as prolonged existence in aqueous mediums due to low rising velocity and large gas–liquid interfacial area, make them highly useful in several applications.

One of the crucial features of MNBs is the generation of ·OH upon their collapse, providing excellent oxidation ability (Atkinson et al., 2019; Liu et al., 2016; Pal et al., 2022). Due to their highly beneficial properties, previous decades have explored the MNBT in numerous applications such as wastewater treatment (Tekile et al., 2017; Wen et al., 2011), potable water treatment, F&V disinfection (US EPA, 1999; Wen et al., 2011), surface disinfection (Tripathi & Hussain, 2022), crop yield improvement (Baram et al., 2022; Xia & Hu, 2018), medical, and health care, whereas many are unexplored yet. The gas decompression type system utilizes sufficient gas to be dissolved in under a suitable pressure to cause a supersaturated condition. Under supersaturated conditions, gas is unstable and escapes from the water generating a large number density of NBs, which follow the Brownian motion and exist in the aqueous solution due to their negligible size (Nirmalkar et al., 2018). MNBO, with its stability and high retention time, reassures about its effectiveness and reaches supersaturation with NBG system, making it more available for outdoor applications. Additionally, MNBs have already been reported for the removal of residual pesticides from F&V surfaces because O_3_ addition in washing solution has been demonstrated to be very effective at breaking down volatile organic compounds (VOCs) like phenols, which are commonly found in pesticide and herbicide residues (Khaled et al., 2018; Lozowicka & Jankowska, 2016).

Previous studies suggested that the size of O_3_ bubbles plays a critical role in pesticide removal within aqueous systems. MNBs significantly influence reaction kinetics within a reactor. Unlike millibubbles or larger macrobubbles (2–5 mm in diameter), which rise rapidly and burst at the water surface due to their low solubility, MNBs remain stable for extended periods beneath the surface (Favvas et al., 2021; Takahashi et al., 2007, 2003). This stability allows MNBs to completely dissolve O_3_ gas in water, making them highly effective for decomposing organic compounds (Ahmed et al., 2018; Ikeura et al., 2011a, 2011b, 2017; X. Li et al., 2024). This section compiles the reported studies showing the importance of O_3_ bubble size for degrading residual pesticides. For example, Ikeura et al. (2011b) explored the use of O_3_ MBs (OMBs) to effectively remove pesticide residues from F&V (Table 3). In their study, MBs smaller than 50 µm were generated through decompression and gas–water circulation systems and tested on lettuce leaves, cherry tomatoes, and strawberries contaminated with fenitrothion (FT) pesticide. The study selected the O_3_‐MBs, O_3_‐millibubbles, and dechlorinated water for comparing their pesticides removal efficiency. The percentage of residual FT in the vegetables was determined, and results revealed that the residual FT removal from lettuce was most effective with the 1.0 mg L^−1^ of O_3_‐MBs (Ikeura et al., 2011b). On the contrary, another study by Ikeura et al. (2011a) showed that FT removal was better with the continuous treatment containing 2.0 mg L^−1^ dissolved O_3_ for cherry tomatoes and strawberries (Ikeura et al., 2011a). The study also demonstrated the difference in the effects of OMBs produced by different methods (Table 3). In the set‐up of this study, an ozone generator was combined with a different MB generator (decompression‐type and gas‐water circulating‐type) to produce OMBs. Like the previous study, the OMB concentration used in this study was 2.0 mg L^−1^, and the vegetables were immersed for 0, 5, and 10 min treatment time. The results revealed that MB generation method has huge impact on the removal efficiency of residual FT because OMBs generated with the decompression type showed better pesticide removal efficiency than that of gas–water circulation type. The reason for the higher efficiency in the decompression‐type OMBs was the half‐life of dissolved O_3_. It was reported earlier that half‐life of O_3_ generated by an air pump in water was 2.27 min in tap water at 25°C, whereas it was 10 min using the gas–water circulation type, and with the decompression‐type half‐life of O_3_ was much longer, which helps to achieve better results than the other O_3_ generation methods. OMBs, after 10 min of the decompression‐type treatment, were able to remove around 16%, 37%, and 56% of FT from cherry tomato, strawberries, and lettuce, respectively (Ikeura et al., 2011a). OMBs, depending on their treatment duration and MB generation method, have shown promising results for removing FT effectively not only from leafy vegetables but also from fruity vegetables. On the contrary, reduction in bubble size to nano scale may lead to improve the residual pesticide removal efficiency due to enhanced AOP.

**TABLE 3:** Effect of different types of O_3_ treatment for the removal of residual pesticides from F&V along with the removal efficiency.

Fruit/crop	Pesticides	Treatment	Ozone Conc.	Time	Removal efficiency (%)	Conclusive remarks	References
Pepper	Malathion Emamectin benzoate Acetamiprid	Tap water	–	10 min	84.80	Remarkable decrease in pesticide residues maintained green color of pepper extend storage life	Özen et al. (2021)
		Aqueous ozone	2 mg L^−1^	10 min	100		
		Ozonated air	2 mg L^−1^	45 min			
		Air	–	45 min	70.08		
	Difenoconazole, fludioxonil, imidacloprid, thiamethoxam	Gaseous ozone	12 µmol mol^−1^	24–48 h	57.9	pAOP treatment combined with gaseous ozone can reduce residual pesticides without greatly reducing quality	Bae et al. (2023)
		Ozone (pAOP) and UV254 irradiation			>50		
	Carbendazim, fluquinconazole, pyrimethanil		24 µmol/mol		<50		
Tomato field soil (treatment under net and green house)	Boscalid Difenoconazole Fludioxonil Pyraclostrobin Tebuconazole	Control	–	40 days	8–15	Ozonized soils showed a decrease in OM content and pH, and an increase in EC and nitrate ion. ozonation‐solarization treatment can solve the RP issue in the soil	Garrido et al. (2023)
		Gaseous ozone	35 g m^−3^ flow rate of 28 L min^−1^		55–61		
Tomatoes	Captan, Thiamethoxam Metalaxyl	Electrical current and ultrasound	1400 mA + 40 kHz	10 min	94.24	EC and US strategies can be considered as effective treatments in industrial scale	Cengiz et al. (2018)
			800 mA + 24 kHz		69.80		
			1400 mA + 24 kHz		95.06		
Soybean	Chlorpyrifos	Cold plasma	1.0–2.0 kV	6 min	50	CP was effective against chlorpyrifos than ozone treatment and caused minor quality changes in soybeans	Anbarasan et al. (2022)
		Ozone treatments	300–550 mg L^−1^	30 min	50		
Lettuce	Methamidophos (MDP), Dichlorvos (DDVP)	Ultrasound and ozone	O_3_ flow rate 75 mg min^−1^	60 min	82.16	O_3_, US and US/ O_3_ had no obvious impact on the quality of the lettuce	Fan et al. (2015)
Tomatoes	Dichlorvos	Tap water	–	15 min	30.7	O_3_ and US are the best of four methods to remove residual pesticides without compromising the tomato quality	Heshmati and Nazemi (2018)
		Detergent	1‐3% sol		91.9		
		Ozone water	2, 4, 6 mg O_3_ L^−1^		70.7		
		Ultrasonication (US)	100–300 V		88.9		
Apple	Captan	Ordinary water	–	30 min	95.0	Post‐harvest ozonation treatment beneficial for quality of fruits	Sadło et al. (2017)
	Boscalid	Ozonated water	2 mg L^−1^	30 min	40		
	Boscalid Pyraclostrobin	Gaseous ozone	10 mg L^−1^		42		
		Ozonated water	2 mg L^−1^	30 min	20		
	Pyraclostrobin Captan	Gaseous ozone	10 mg L^−1^	30 min	32		
		Gaseous ozone			78.89		
Undamaged Apple Damaged Apple	Captan Difenoconazole Linuron	Gaseous ozone Ozone in gas (5 mg L^−1^)	Optimized to 1 mg L^−1^	12 h for 1 min up to 84 days	70.64	Ozone slowed down the ripening of apples hence increasing the shelf life	Antos et al. (2018)
					>95		
Carrots	Quality test	Gaseous ozone fumigation	5.5 g h^−1^	60 min	–	O_3_ as gas and dissolved in water did not alter the weight loss %, firmness and the color of carrots. Improved the shelf‐life	Souza et al. (2018)
		Ozonated water	0–10 mg L^−1^		–		
Chili	Malathion	Gaseous ozone fumigation	5.5 g h^−1^	30 min	68	Ozone fumigation proved to be a rapid method to decay the pesticide residue in dried chilies	Sintuya et al. (2018)
	Chlorpyrifos				51		
	Profenofos				45		
	Ethion				66		
Tomato	Myclobutanil (*C*_0_: 2, 6 10 mg L^−1^)	Aqueous ozone	Tap water	15 min	12.37–15.29	Reduction of residual pesticides removal from F&V is concentration–time de‐pendent	AlAntary et al. (2018)
			0.5 mg L^−1^		82.60–92.31		
			2 mg L^−1^		88.82–96.56		
			5 mg L^−1^		94.80–98.32		
Kumquat	Chlorpyrifos	Ozone solution	0.4 mg kg^−1^	30 min	24	16 pesticides were removed by ozonation at a certain level but not very effective	Wu et al. (2019)
	Myclobutanil				50		
	Tebuconazole				63		
	Bifenthrin				50		
	Lambda‐cyhalothrin				61		
	Beta‐cypermethrin				49		
	Esfenvalerate				44		
	Difenoconazole				44		
	Acetamiprid				42		
	Imidacloprid				33		
Cucumber	Above 10 pesticides				17–40		
Spinach					53–78		
Table grape	Chlorothalonil	Aqueous ozone	2.0 mg L^−1^	60 min	60	Treatment at 2 mg L^−1^ ozone maintained fruit quality for a longer storage period while 3.0 mg L^−1^ may alter the taste and quality	Heleno et al. (2015)
Strawberry	Chlorpyrifos	Aqueous ozone	1.0 mg L^−1^	5 min	75.1	US cleaning and boiling were effective treatments then the ozone water	Lozowicka and Jankowska (2016)
					36.1		
	Tetraconazole	US treatment	Frequency 40 kHz, power 2 × 240 W peak/period		79.1		
					84.5		
*Brassica* sp.	Diazinon	ozonated water	1.4 mg L^−1^ at 24°C,	30 min	53.4	Ozonation is a safe and promising treatment for domestic use without compromising the quality of F&V	Wu et al. (2007)
	Parathion				55.3		
	Methyl‐parathion				47.9		
	Cypermethrin				61.1		
Tomato	Mancozeb	Chlorine	100 mg L^−1^	20 min	71	Because of the instability of the O_3_ gas a great proportion of dissolved ozone would escape or reduce to oxygen molecules in a few minutes hence, ozone treatment did not show effectivity for mancozeb removal	Cengiz and Certel (2014)
		Hydrogen peroxide	100 mg L^−1^		65		
		Ozone	3 mg L^−1^		60		
**Micro/Nanobubble ozone treatment**							
	Fenitrothion (FT)	Ozone microbubbles	1.0 mg L^−1^ OMCBs	10 min	51	OMCB solutions are more effective than OMLB and tap water	Ikeura et al. (2011b)
			2.0 mg L^−1^ OMCBs		55		
			2.0 mg L^−1^ continuous bubbling of OMCBs		58		
Cherry tomatoes			1.0 mg L^−1^ OMCBs		10		
			2.0 mg L^−1^ OMCBs		10		
			2.0 mg L^−1^ continuous bubbling of OMCBs		35		
Strawberry			1.0 mg L^−1^ OMCBs		10		
			2.0 mg L^−1^ OMCBs		15		
			2.0 mg L^−1^ continuous bubbling of OMCBs		25		
	
Lettuce	Fenitrothion (FT) 500 mg L^−1^	Gas‐water circulating‐type	2.0 mg L^−1^	10 min	54.76	The decompression type was more effective than the gas‐water circulation type	Ikeura et al. (2011a)
Cherry tomato					8.9		
Strawberries					14		
Lettuce		The decompression type			56.29		
Cherry tomato					16.22		
Strawberries					37.59		
Apple	Trichlorfon	Tap water	–	10 min	77.4	The efficient removal of pesticides is related to the surface matrix components of F&V. OMBT has little effect on the surface color of apples	Li et al. (2021)
	Carbosulfan				87.5		
	Trichlorfon and carbosulfan	Hypochlorous acid‐water	40–80 mg kg^−1^ available chlorine	5 min	60–70		
		Ozone‐microbubble treatment (OMBT)	Flow rate 0.33 L s^−1^		98–100		
		ozone‐water	2.5 mg L^−1^		86–88		
		Microbubble water (MCB)	0.33 L s^−1^		90–91		
Strawberries	Emamectin Benzoate Azoxystrobin Boscalid Difenoconazole	Ozone micobubble	OH radicals 8.9 and 10.2 µmol L^−1^	18 min	51–65	Ozone can decompose to produce ⋅OH in water, with the dissolution rate comparable to its dissipation rate at 0.67 s, ozone proved to be a best method to maintain the quality of F&V	Li et al. (2024)
Cherry					51–59		
Apricot					24–70		
Parsley	Quality testing	O_3_‐MNBW	2.5 mg L^−1^	10 min O_3_‐MNBW before 5 days storage	–	O_3_‐MNBW treatment effectively preserved the sensory quality of parsley. A higher level of firmness, vitamin C, and chlorophyll content was tested in the treated samples	Shi et al. (2023)

**FIGURE 6:**
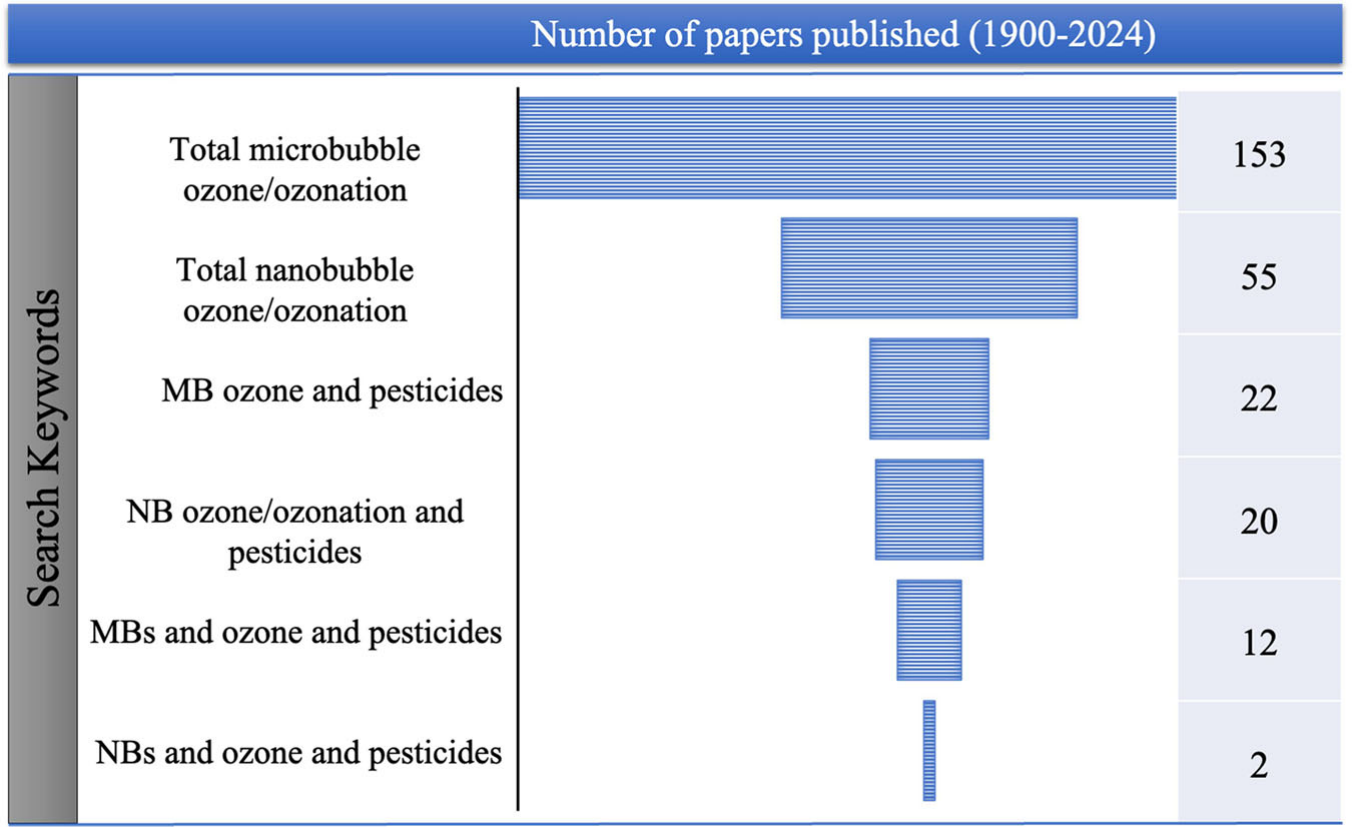
Statistics obtained for number of papers published on micro‐ and nanobubble (MNB) treatment for pesticides removal. *Source*: Web of science.

Li et al. (2021) investigated TCF and carbosulfan residues removal from apple surfaces by various washing approaches such as washing with tap water, hypochlorous acid (HClO)‐water, O_3_‐water, MB water, and OMB treatment. The study found that O_3_‐water, MB water, and OMB treatment represented significantly (*p* < .05) higher removal rates than tap water and HClO‐water (Table 3). Ozone‐water, MB water, and ozone‐MB treatment for 5 min have demonstrated remarkable removal rates of 86%–88%, 90%–91%, and 98%–100%, respectively, whereas the other two methods also showed 88%–100% removal of carbosulfan, and TCF was 77%–100% within 10 min. As O_3_ treatment has shown its effect within 5 min, it is considered best because it is less time consuming. The synergistic effect was noted when O_3_ is coupled to MB water, resulting in a 100% reduction of pesticide from the apple surface. The effect might be explained by the oxidation of O_3_, which creates high levels of OH·, and when there is contact between pesticide residues and OMB treatment, pesticides degrade faster than other treatment methods (Ikeura et al., 2011a, 2011b; C. Li et al., 2021). Another study compared domestic washing methods (water and detergent) and ozone treatments (ozone bubbling and ozonated water) for removing fungicide residues, namely, azoxystrobin, chlorothalonil, and difenoconazole from tomatoes. Results concluded that ozone bubbling at 3.0 mg L^−1^ was most effective, reducing residues by 70%–90%, but caused more fruit mass loss during storage compared to lower ozone concentrations (Rodrigues et al., 2019). Total number of papers published in this area is presented in Figure 6, which also indicates the need to carry out research in applications of MNBT in the agriculture field.

## 3 EFFECTIVE FUNCTIONAL ROUTES AND REACTION MECHANISM OF OZONE FOR POLLUTANTS DEGRADATION

**FIGURE 7:**
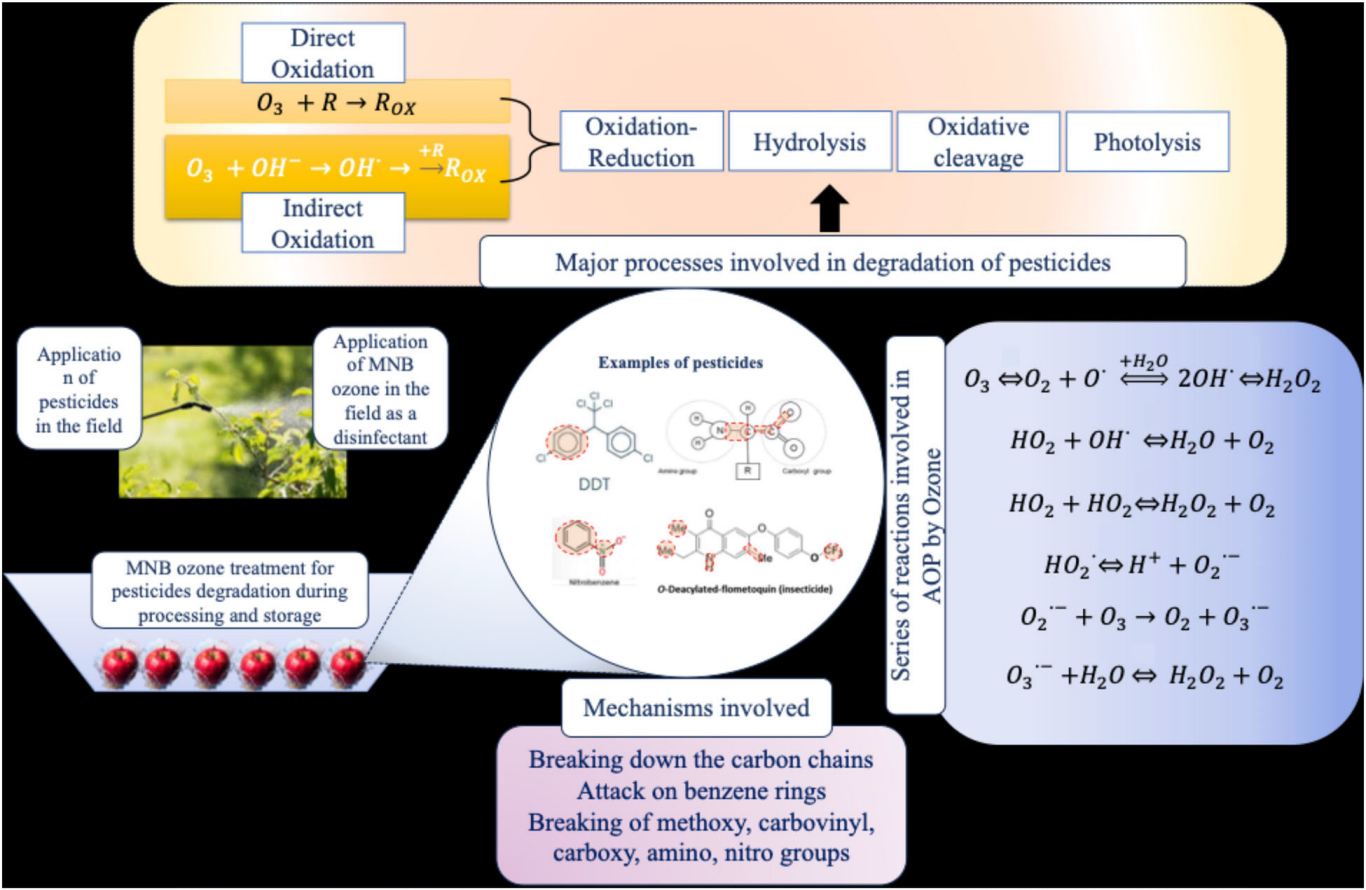
Major processes, reactions, and mechanisms involved for the effective degradation of pesticides in aqueous medium by micro‐ and nanobubble (MNB) O_3_.

An O_3_‐mediated process for the degradation of organic matter or any contaminants involves two types: direct and indirect reaction mechanisms. Among the two systems, one dominates, whereas both the oxidations will take place in the same system. When O_3_ encounters the pesticides to initiate the breakdown of pesticides in the MNBO system, radicals attack the functional groups of pesticides such as amino, methoxy, dichlorovinyl, nitro, alkanes, and alkynes. Due to such structural hinderance, pesticides lose efficiency due to oxidative cleavage (Aidoo et al., 2023). Such degradation which is selective toward specific compounds and functional groups is called direct reaction of O_3_. It is usually initiated when O_3_ self‐decomposes and produces a stable oxygen molecule (O_2_) and an activated atom of oxygen (O·) (Figure 7) (Flanagan, 2021; Jamali et al., 2024). Although in the indirect reaction, the spotlight is on the ^•^OH radicals produced from O_3_ decomposition in multiple steps or secondary oxidation, produced resultants such as O·, OH·, O_3_·, and H_2_O_2_ are instrumental in degrading the pesticides (Figure 9). This reaction is very fast, complex, and largely nonselective (Chen et al., 2024; Flanagan, 2021). In an aqueous system, pesticides can be degraded mainly by hydrolysis, photolysis, and reduction–oxidation reactions. When light falls on the molecule, it helps the excitation of the molecule from the ground state to the excited state, and when it comes back to the ground state, it releases the energy, being responsible for indirect photolysis (Aidoo et al., 2023; Pirsaheb & Moradi, 2020). The resultant reactions are isomerization, decarboxylation, carbon oxidation, dehalogenation, ester cleavage, cyclization, or sulfide oxidation (Aidoo et al., 2023). Pesticides, when degraded and O_3_ reacts with these unsaturated carbon chains, result in forming acids, alcohols, amines, carbonyls, carbohydrates, and carboxylates; hence, pesticides can be rinsed away with water. As O_3_ is highly reactive and fast degraded in water, MNBO may overcome the fast self‐degradation because of high mass transfer and abundantly available nanoentities to provide enough dissolve O_3_ for residual pesticide degradation. To date, only a few studies are available showing the effect of O_3_‐MNBs for preserving the quality of crops (Shi et al., 2023) or for reducing the pesticides from F&V (Ikeura et al., 2011b). However, the potential of O_3_‐MNBs in pesticide removal from F&V is a promising area that requires further exploration. The application of NBO may enhance the efficiency of degradation manifolds because of the presence of a large number of nanoentities. There is a possibility that the reaction of NBO is much faster than the MB ozone due to generation of a high number of ·OH radicals within the short time.

### 3.1 Factors affecting the degradation of pesticides by ozone

The reaction in aqueous phase or on the F&V surface depends on temperature, pH, and chemical composition of the water (Flanagan, 2021). However, the degradation efficiency by O_3_ is influenced by factors like O_3_ concentration, the generation of ROS, and the mass transfer of O_3_ from the gas phase (gaseous O_3_) to the liquid phase (aqueous O_3_). To analyze the mass transfer between gas–liquid interfaces, the two‐film theory is used, which suggests that the mass transfer of O_3_ in liquid is influenced by the distinct behaviors of gaseous and aqueous O_3_. Theoretically, mass transfer occurs through two films where both the films act as resistance barriers: the gas film adjacent to the liquid interface and the liquid film adjacent to the gas interface. The concentration gradients within each film determine the rate of mass transfer (Han et al., 2022; John et al., 2022). The overall mass transfer rate is governed by the resistances in both phases and can be expressed by the Equation (1), and O_3_ utilization can be calculated by Equation (2) (Xiong et al., 2019):

(1) $$\mathrm{Rate}\;\mathrm{of}\;\mathrm{mass}\;\mathrm{transfer}=\frac{C_{\mathrm{gas}-}C_{\mathrm{liq}}}{R_{\mathrm{gas}}+R_{\mathrm{liq}}}$$

(2) $$\begin{array}{ccc} & & O_{3\;}\mathrm{utilization}\;\left[\%\right]\\ & & \;=\frac{O_{3}\left(\mathrm{input}\right)\left[\mathrm{mg}\;L^{-1}]-O_{3}\left(\mathrm{off}\;\mathrm{gas}\right)[\mathrm{mg}\;L^{-1}\right]}{O_{3}\left(\mathrm{input}\right)\left[\mathrm{mg}\;L^{-1}\right]}\times 100 \end{array}$$

where *C*_gas_ and *C*_liq_ are the concentration gaseous and aqueous O_3_, respectively. *R*_gas_ and *R*_liq_ are the corresponding resistances to mass transfer. *R*_gas_ could depend on factors like the diffusion coefficient or the boundary layer thickness in the gas, and *R*_liq_ could be dependent on properties like diffusion coefficients or boundary layers in the liquid phase (Tanaka et al., 2020).

For gaseous O_3_, mass transfer into the liquid is driven by the concentration difference in the gas phase, and its diffusion rate influences the efficiency of dissolution (Tanaka et al., 2020). In the form of millibubbles or MBs, however, O_3_ may decompose faster under sunlight, which limits its availability for transfer to the liquid. As in aqueous systems, O_3_ solubility is low, and its dissolution competes with reactions that produce ROS further affect ozone availability. Micro‐nano‐aqueous ozone benefits from a larger surface area, enhancing dissolution and ROS generation, hence more available for degradation of pollutants. Concentration gradients, temperature, pH, and the presence of surfactants or MNBs can be the key factors that affect the ozone dissolution in aqueous medium. MNBs, in particular, increase the gas–liquid interfacial area, improving mass transfer efficiency by stabilizing O_3_ and slowing its decomposition, leading to enhanced pesticide degradation. In MNBO systems, the small size and slow rise velocity of MNBs further enhance the contact time between O_3_ and pesticide molecules, improving pesticide degradation efficiency (Xiong et al., 2019). By optimizing these conditions, ozonation systems can achieve more effective and sustained removal of pesticide residues from F&V, offering a powerful solution for pesticide degradation in both open and closed systems.

## 4 ENVIRONMENTAL ASPECTS OF USING MNBO FOR RESIDUAL PESTICIDE REMOVAL

In agriculture, MNBs find utility in water treatment for irrigation, effectively eliminating pathogens and contaminants without leaving harmful residues (Pal et al., 2022). It enhances soil health by reducing microbial loads, suppressing diseases, and promoting nutrient availability. For instance, Ito and Sugai (2021) reported that CO_2_ and O_2_ NBs resulted in enhance growth of *Pseudomonas aeruginosa* by acting as a ferrous ion carrier and as the oxygen source, respectively (Ito & Sugai, 2021). O_3_’s oxidative properties also enable it to serve as an eco‐friendly pesticide alternative as it is a natural disinfectant. In sanitation, O_3_’s potent disinfectant properties are widely employed, efficiently eradicating bacteria, viruses, and fungi. This makes it invaluable for sterilizing water, surfaces, and air in various settings, including hospitals and food processing facilities. As far as food items are concerned, if we use MNBO to clean the raw material, we can ensure raw material purity and user safety. Studies have shown the effectiveness of ozonation in controlling diseases, but no experimental study is available on using NBO as a disinfectant or spray before harvest or soil amendments. However, several studies reported that, apart from treating F&V with MNB ozonation for residual pesticide removal, remediation of pesticide‐contaminated soils is possible using ozonation combined with solarization. For instance, Garrido et al. (2023) carried out the experiment in two agricultural areas of greenhouses dedicated to growing tomatoes under net systems. O_3_ treatments were applied using a piped network, both on the soil surface and subsurface, delivering O_3_ in a gaseous form. Treatment time was forty (40) days; the average pesticide degradation reached approximately 55%–61% for both farming systems, whereas the control soils exhibited only an 8%–15% reduction in residual pesticides levels. The impact of ozonation‐solarization on soil physical–chemical properties was also evaluated. The results suggested that combining ozonation with solarization techniques could be a viable approach for remediating pesticide‐contaminated agricultural soils (Garrido et al., 2023). In another study, Martínez‐Escudero et al. (2022) used ozonation and solarization for the eradication of pesticide residues such as anilinopyrimidine, triazole, neonicotinoid, and strobilurin in agricultural fields and degraded the main metabolites (found main 15 transformation products) under greenhouse conditions. This study was mainly done to demonstrate the effect of temperature and O_3_ application, which showed that solarization together with deep ozonation resulted in increased soil temperature, providing the best results (Martínez‐Escudero et al., 2022). Díaz‐López et al. (2021) reported that ozonation greatly affects soil's physicochemical properties. Moreover, ozonation can influence or alter the composition of soil microbial community, activity, and total biomass. It was evident from their results that the fungal biomass and its composition were more sensitive to O_3_ than the bacterial biomass when exposed to solarization and ozonation in combination. Although O_3_ is known for its strong bactericidal properties, relative abundances of several pesticide‐degrading bacteria increased, and their pesticide degradation efficiency improved in the soil system. Overall, decrease in fungal community and in *Fusarium* (typical pathogen) was observed due to solarization. On the other hand, potential pesticide degraders, such as *Aspergillus* and others like *Cladosporium* and *Rhizopus*, were increased by O_3_ treatments (mainly the SOS (surface treatment). It was demonstrated that combined ozonation and solarization are better for removing pesticides from soil. The degradation of the pesticides after 50 days of treatment was enhanced by 20%, 28%, and 33% in solarized soil, solarized soil with surface ozonation (SOS), and solarized soil with deep ozonation, respectively, in comparison to untreated soil (Díaz‐López et al., 2021).

O_3_ NBs have recently been shown to reduce pathogens, biochemical oxygen demand (BOD), chemical oxygen demand (COD), total soluble solids (TSS), and improve dissolved oxygen (DO) level in pond water without influencing the fish innate immunity (Huang et al., 2023). Pal et al. (2022) reported the case study of four different sites in India for the efficiency of NBO to improve the pond water quality. Experiments were performed on‐site as well as off‐site, and it was found that NBO was successfully able to reduce 55%–82% of COD, 63%–91% reduction in BOD, and 83%–99% reduction in TSS at all the four sites. Free ammonia was reduced by 62%, and DO level was drastically improved and reached up to supersaturation level of 26.5 mg L^−1^ in some cases. DO remained stable for days at all the sites and reduced very slowly because of the presence of oxygen in the form of NBs (Pal et al., 2022). However, there is not much information available about effect of NBs on the microbial community of ponds. Recently, Huang et al. (2023) investigated the impact of O_3_ macrobubbles and NBs on the microbial ecology of pond water and fish health and were able to successfully eradicate the 90.9%–99.4% of the heterotrophic bacteria and eliminated around 95.2% of the bacterial DNA from pond water ecosystems by treating with the 0.15 mg L^−1^ ozone (Huang et al., 2023). This solution is chemical‐free, long‐lasting, and sustainable in maintaining the water quality, sanitization, and surface disinfection (Gonçalves & Gagnon, 2011; Pal et al., 2022; Powell & Scolding, 2018). Overall, we can emphasize that reducing pesticide use through innovative agricultural technologies such as MNBO offers significant environmental benefits. Minimizing chemical runoff into water bodies will help preserve water quality and aquatic ecosystems. Improved soil health is another advantage, as reduced pesticide applications foster healthier soil microbial communities and enhance fertility (Díaz‐López et al., 2021). Decreased air pollution from VOCs and aerosols further contributes to cleaner air and improved human health. Wildlife can be protected by reducing the levels of pesticides in the environment, and the risk of bioaccumulation and biomagnification can also be reduced. MNBO is one of the sustainable agricultural practices that enhance resilience to climate change impacts and promote water and soil conservation.

## 5 IMPACT ON THE COUNTRY'S ECONOMY ADOPTING MNBT

As mentioned before, the overuse of pesticides presents a multitude of challenges, including trade barriers, health issues, environmental concerns, and livestock. The direct and indirect costs of excessive pesticide (Bourguet & Guillemaud, 2016; Pimentel, 2005) can be mitigated by the widespread adoption of MNBT. Many countries follow stringent regulations on pesticide residues in imported food products to protect consumer health. Failure to meet these standards can result in the rejection of shipments, leading to delays, additional testing costs, and potential loss of market access. Residue limits act as a market entry barrier for exporters because stricter limits for importers result in an average reduction of 8.8% in the trade of F&V (Hejazi et al., 2022). Significant economic losses can occur for exporting countries from the rejected shipments. These losses include the cost of the rejected goods, transportation, storage, and potential fines or penalties imposed by importing countries, decreased demand, loss of market share, and difficulties in maintaining long‐term trade relationships. Studies indicate that agricultural exports affected by pesticide residue issues can significantly reduce a country's gross domestic product growth.

**FIGURE 8:**
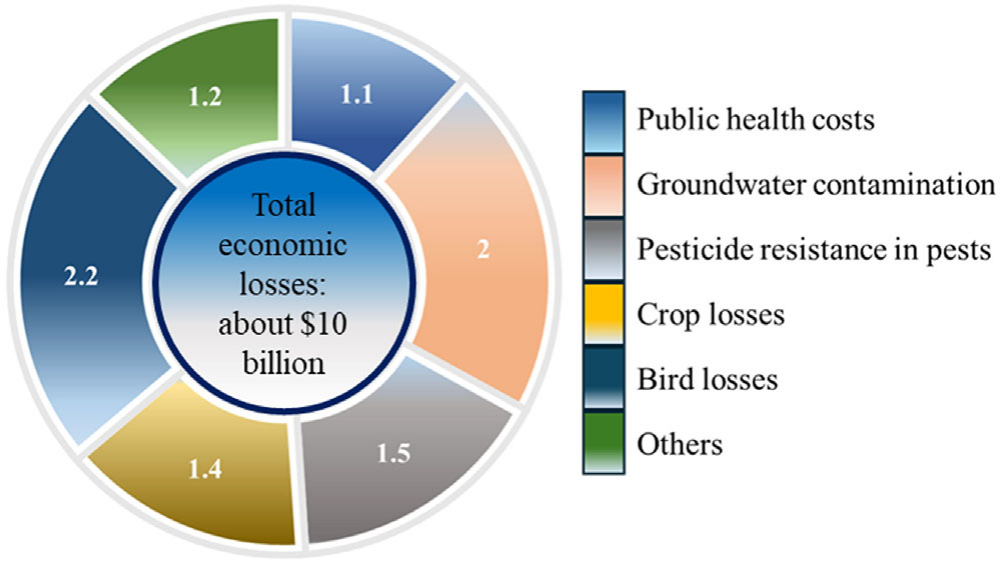
Summary of the total economic losses and their contribution in social, health and environmental losses in the USA, as per the report of Pimentel (2005).

Bourguet and Guillemaud (2016) analyzed that the cost associated with each 1% of cancer due to pesticides is approximately 20 billion dollars annually. According to Pimentel's (2005) report, the major direct and indirect economic and environmental losses occurred in the USA due to pesticide application estimated a total of $10 billion which was $8 billion in 2001 (incomplete analysis) (PAN Europe, 2005; Pimentel, 2005). The above costs also include social damages such as $1.1 billion annually in public health costs and $2.0 billion resulting from groundwater contamination. Pesticide resistance in pests, which is a serious concern now, costs around $1.5 billion and $1.4 billion in crop losses due to excessive use of pesticides along with $2.2 billion in bird losses (Figure 8) (Pimentel, 2005). Such problems can be tackled if we have a technology which is universally‐applicable such as O_3_‐MNB, to remove the residual pesticides or for safe storage (Antos et al., 2018; Rodrigues et al., 2019). Japan is the leading country to adopt MNBO for different applications, including the development of industrial scale ozone generators for washing and disinfecting F&V using MNBO technology to remove pesticides, post‐harvest cleaning in packhouses, and water treatment. Other countries to adopt the MNBO are South Korea, United States, China, Germany, and Australia.

## 6 CHALLENGES AND PROSPECTS OF RESEARCH ON MNB USAGE IN THE FOOD INDUSTRIES

**FIGURE 9:**
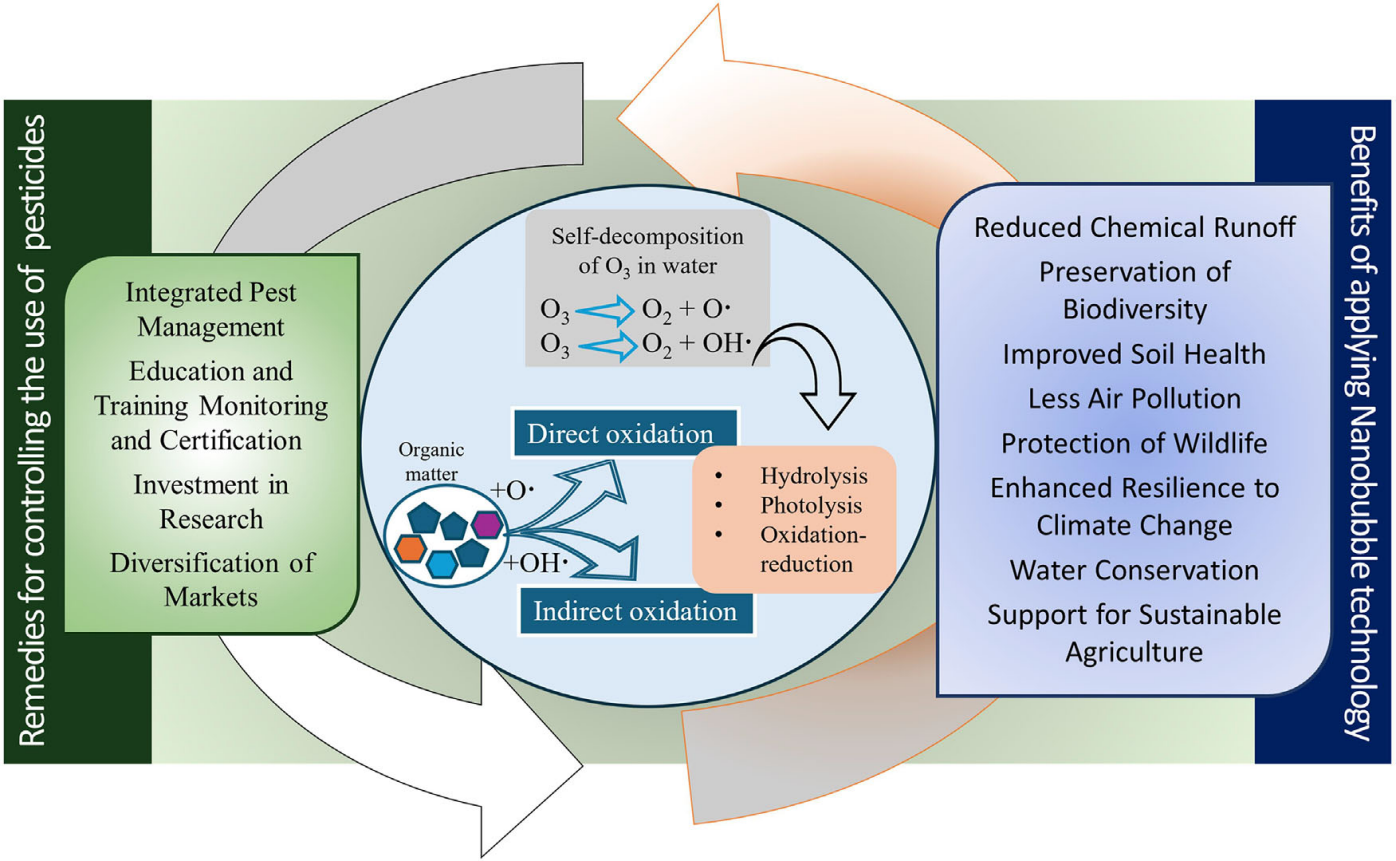
Benefits of applying remedies and micro‐ and nanobubble (MNB) ozone for the sustainable environment.

Figure 9 elucidates the benefits of adapting MNBT along with the pest management practices in agriculture to reduce the overall use of pesticides. Despite mind‐blowing qualities and contribution of MNBO in the field of agriculture and food industries, the development and commercialization of ozonation MNB processing plants face several key challenges; high capital investment is the biggest one. As NBT is still in its nascent phase, the generation of MNBs needs a continuous monitoring, and precise control of O_3_ dosage, flow rates, and contact time requires sophisticated equipment and continuous monitoring. Energy consumption involved in MNBO and the production of stable nano‐bubbles is quite high, which can be inefficient compared to traditional methods. Research into more energy‐efficient O_3_ generation methods and optimizing all the parameters to stabilize and maximize the dissolution and availability of O_3_ for pesticides degradation could reduce operational costs. Additionally, this will help to automize and improve the efficiency of ozonation systems. Furthermore, integrating sensor technologies to monitor ozone concentration, ROS formation, and pesticide degradation in real‐time could lead to more precise control of the ozonation process, enhancing both efficacy and cost‐effectiveness. Furthermore, collaboration between research institutions and industries can drive cost‐effective scaling and better system integration for larger commercial plants. The mechanism of pesticide degradation via ROS generated from MNBs remains poorly understood, particularly in terms of the reaction kinetics between nanosized O_3_ and different pesticide classes under real‐world conditions (Xiong et al., 2019). This could involve exploring different pesticide categories, system parameters, and incorporating advanced modeling techniques to predict the outcomes.

Regulations and policies on the safe usage and exposure levels of O_3_ in packhouses with the proper ventilation and monitoring systems to ensure worker safety are necessary for the adoption of the such new technologies. In response to these regulatory concerns, an alternative to ozone could be H_2_O_2_ which is effective in pesticide degradation and sterilization but has less stringent safety concerns compared to ozone (Aidoo et al., 2023). However, more research and testing are required to determine the effectiveness, cost, and scalability of these alternatives in comparison to ozonation, especially in industrial‐scale applications like packhouses.

## 7 CONCLUSIONS

O_3_ has been used as a food sanitizer and surface disinfectant for a long time and is also used in water treatment. MNBT emerges as a promising solution for addressing several issues that were the bottleneck of using O_3_ in its usual form, such as instability, low dissolvability, and cost. It helps tackle global issues like climate change and environmental sustainability, reducing costs and energy consumption in industries, optimizing therapeutic and diagnostic techniques, and various other applications. This review covers recent advancements in washing and sanitizing F&V, especially by ozonation, with detailed insights into the effect of bubble size on the O_3_ gas dissolution in aqueous medium and its uses for residual pesticides degradation. It will be helpful in eradicating the health issues, quality issues, and rejection issues associated with the pesticides. Environmental damage concerns will also be solved by using this natural gas as a disinfectant. Economic losses due to the rejection of exports, social losses, and environmental losses can also be mitigated by using MNBO. Applying MNBs will improve yield and potentially save significantly the fertilizer/herbicides/other chemicals used in the field. Despite the promising potential of MNBO for residual pesticide removal further exploration and studies in MNBO are necessary to fully assess and optimize the environmental and economic benefits of these hybrid technologies. On the other hand, integrated pest management practices can help reduce reliance on pesticides by promoting natural pest control methods, crop rotation, and the use of resistant varieties, as well as implementing rigorous monitoring systems and certification processes to ensure compliance with international standards on pesticide residues. Encouraging investment in research and innovation is crucial for developing new technologies and implementing those in the agriculture practices. This can lead to a safer and more effective alternatives, such as biopesticides and MNBO. However, it is essential to mention that governmental support is indispensable in bridging these novel technologies to the market. Financial assistance, subsidies, and incentives can help farmers adopt sustainable agricultural practices. This review paper advocates not only adopting advanced technologies, regulations, and policies for O_3_ usage in the industries and in packhouses. More support of government could help people to accept and adopt new technologies and methods for pesticides management and control to address the challenges through sustainable practices and adherence to international standards.

## AUTHOR CONTRIBUTIONS

**Preeti Pal**: Conceptualization; methodology; investigation; validation; funding acquisition; writing—original draft; writing—review and editing; visualization. **Arata Kioka**: Funding acquisition; writing—review and editing; project administration; resources; visualization.

## CONFLICT OF INTEREST STATEMENT

The authors declare no conflicts of interest.
